# Atypical hepatic haemangiomas

**DOI:** 10.1259/bjrcr.20170029

**Published:** 2017-11-01

**Authors:** Juan David Vásquez Montoya, Beatriz Molinares, Elsa María Vásquez Trespalacios, Vanesa García, Juan Camilo Pérez Cadavid

**Affiliations:** ^1^Department of Radiology, Universidad CES, Medellín, Antioquia, Colombia; ^2^Department of Radiology, Pablo Tobon Uribe Hospital, Medellín, Colombia; ^3^Department of Radiology, Universidad de Antioquia, Medellín, Antioquia, Colombia; ^4^**Radiology**, Pablo Tobón Uribe y Dinámica IPS Hospital, Medellín, Colombia

## Abstract

Hepatic haemangioma is the most common benign liver lesion in the general population. It often exhibits a uniform pattern of characteristics, thus being called “typical.” However, a certain number of hepatic haemangiomas have special or uncommon characteristics and are termed “atypical.” The majority of patients are asymptomatic. Its differential diagnosis is critical, and its differentiation from other aetiological possibilities can be challenging, especially in cases of atypical haemangiomas, which may lead to confusion or even misleading diagnoses. We report on a 55-year-old patient with atypical multiple hepatic haemangiomas mimicking metastasis or echinococcus infection.

## Introduction

Hepatic haemangiomas are benign non-neoplastic hypervascular liver lesions. According to newer nomenclature, these lesions are known as slow flow venous malformations. We retain in the article the word haemangiomas, as this term is ubiquitous in the literature and familiar to most clinicians and also to be consistent with the majority of the existing literature.

Most haemangiomas present with typical and common characteristics in the general population, but those termed “atypical” occur less frequently. The spectrum of atypical haemangiomas is important and can help one avoid most diagnostic errors. However, in some cases the diagnosis will remain uncertain at imaging, and will require histopathological examination. The case presented exhibits the presence of multiple lesions that make diagnosis difficult, owing to similarity with other relevant differential diagnoses, such as metastases or hepatic lesions from echinococcus. We consider it important to publish this case because of its peculiar characteristics.

## Clinical case

A 55-year-old female patient with a history of systemic lupus erythematosus was admitted to the haematology external consultation service as a result of a clinical picture of fatigue and constitutional syndrome of 5 months’ duration.

**Figure 1.**
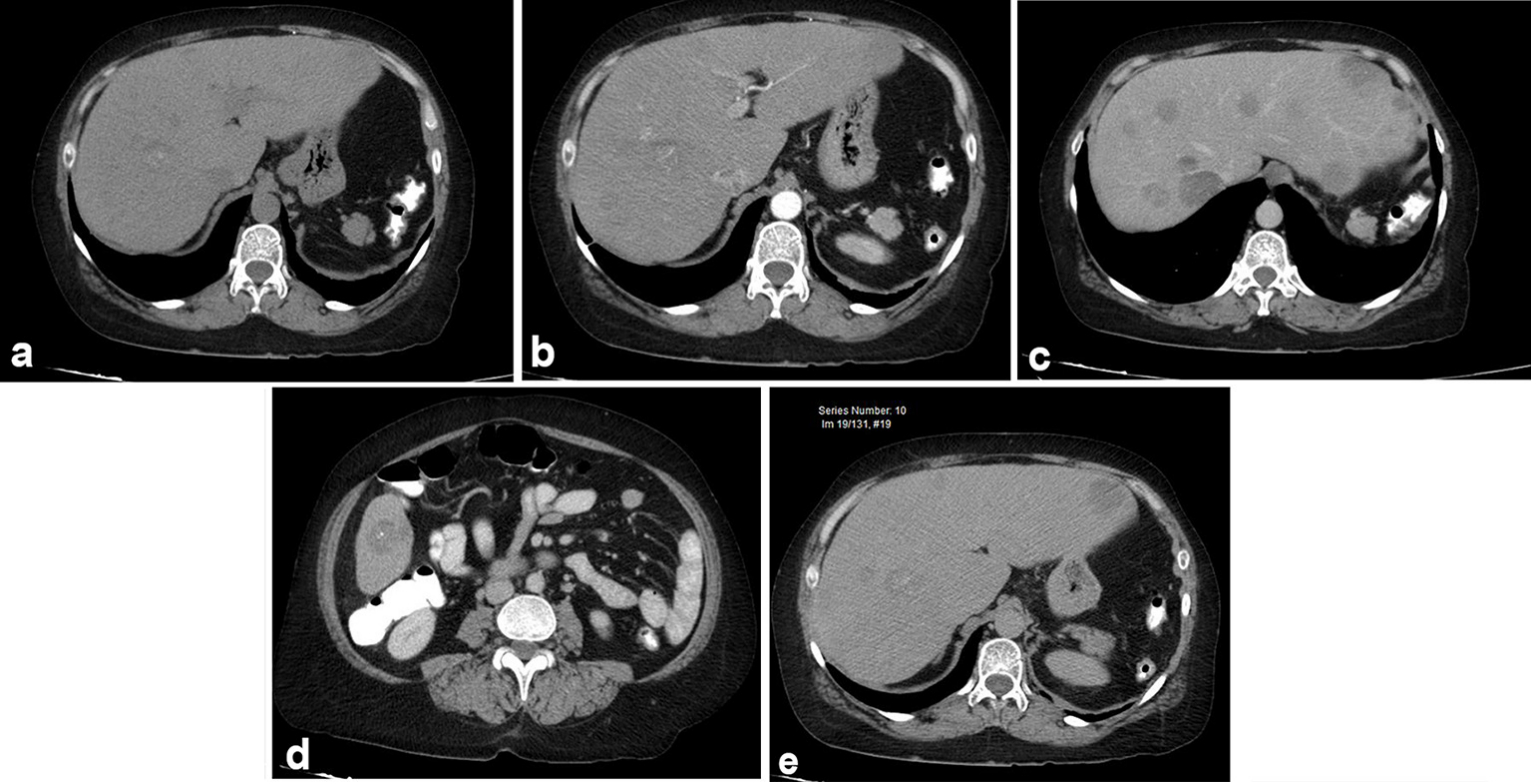
Abdominal CT showing multiple hepatic lesions ranging from a few to 5 cm, which do not significantly enhance and contain microcalcifications. (a) Simple phase: focal lesions are hypodense with microcalcifications (arrow); (b) arterial phase, without significant enhancement: no hypervascular lesions are identified. (c and d) Portal phase: discrete uptake; (e) late phase: persisting hypodense lesions without retention of contrast medium.

She presented with an extrainstitutional CT that demonstrated multiple focal, hypovascular liver lesions with microcalcifications (Figure 1) that occupied all segments of the liver, and was interpreted as possible metastatic disease.

The patient was evaluated without positive findings upon physical examination. The extrainstitutional paraclinical tests showed negative tumour markers and normal tests of liver function.

**Table:** 

Paraclinical studies			
Alpha fetoprotein	3.73	Direct bilirubin	<0.10
**Carcinoembryonic antigen**	3.06	Alkaline phosphatase	114
Ca 19-9	0.6	Gamma- glutamyltransferase	159
Lactate dehydrogenase	186	Aspartate transaminase	31
Ca 125	29	Alanine aminotransfe rase	21
**HBsAg**(hepatitis B surface antigen)	0.19	prothrombin time	11.9
Total bilirubin	0.33	INR	1.09
**Hemoglobin**	14.4	Neutrophils	58.2
Hematocrit	44.2	Lymphocytes	36.4
Red blood cells	4.66	Basophils	0.3
Mean corpuscular volume	94.8	Eosinophils	4.3
Mean corpuscular hemoglobin	30.8	Monocytes	0.8
Mean corpuscular hemoglobin concentration	32.4	Platelets	243000
Red blood cell distribution width	15.4	Mean platelet volume	9.2
White Blood Cells	12000	Anisocytosis	+

Because of the imaging findings, it was decided to hospitalize the patient to characterize the hepatic lesions with contrast MRI with gadopentetate dimeglumine (Magnevist), 15 ml, infusion rate 2.2 psi, to stage the possible tumour lesion with a study algorithm of an unknown primary tumour. No specific liver contrast medium was used.

**Figure 2.**
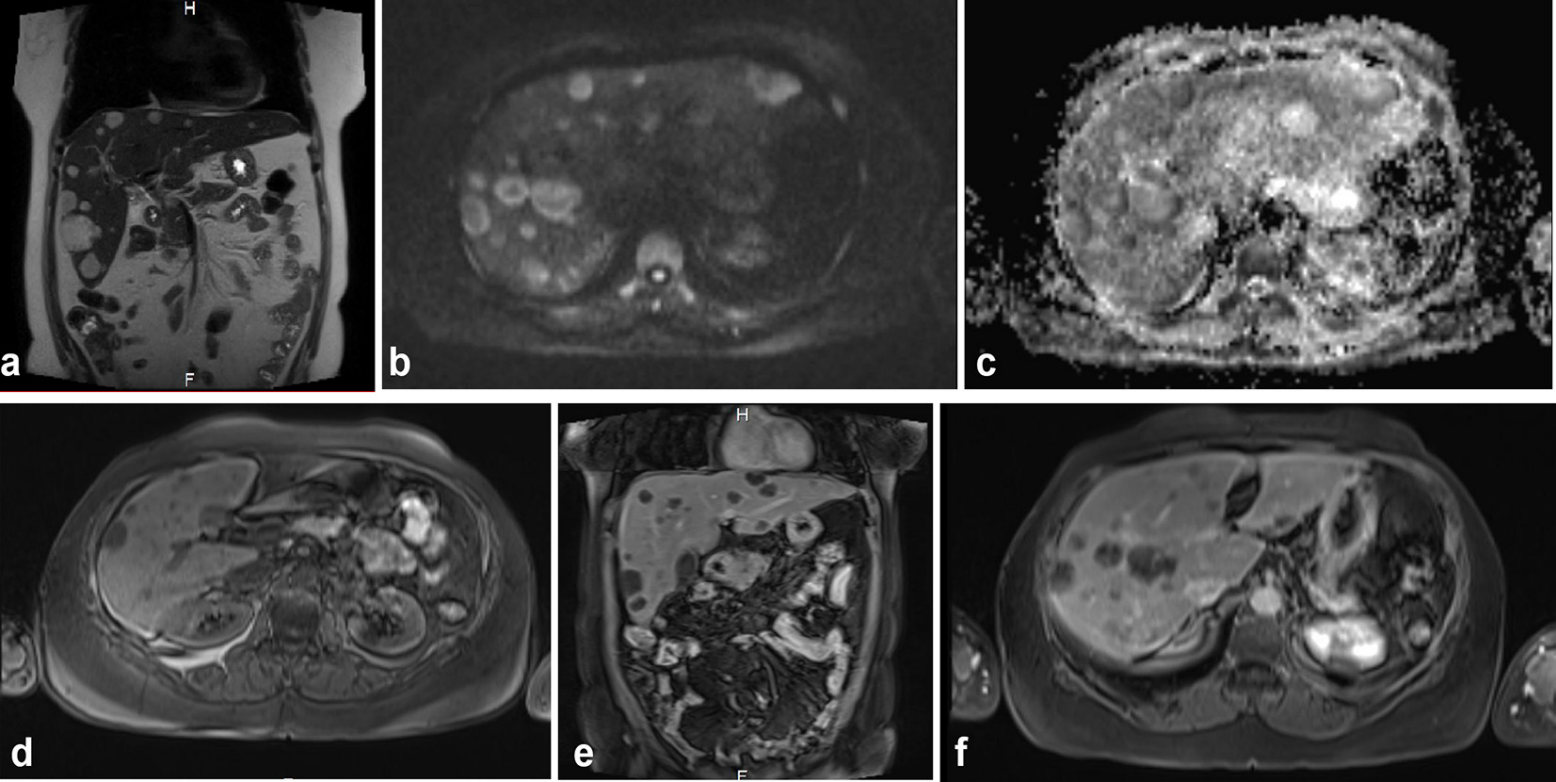
MRI showing a similar pattern of enhancement. (a) Coronal sequence, *T*_2_ Half Fourier Single Shot Turbo-spin Echo, (HASTE): normal-sized liver with multiple, hyperintense lesions in all liver segments; (b and c) diffusion sequences: lesions show restriction predominantly in the periphery; (d) *T*_1_ fat suppression without contrast media: hypointense lesions in the liver; (e) *T*_1_ coronal sequence in portal phase: the lesions remain without significant uptake of contrast medium; (f) Axial *T*_1_ late phase, 5 min after administration of contrast medium: the lesions with some small zones of central uptake.

**Figure 3.**
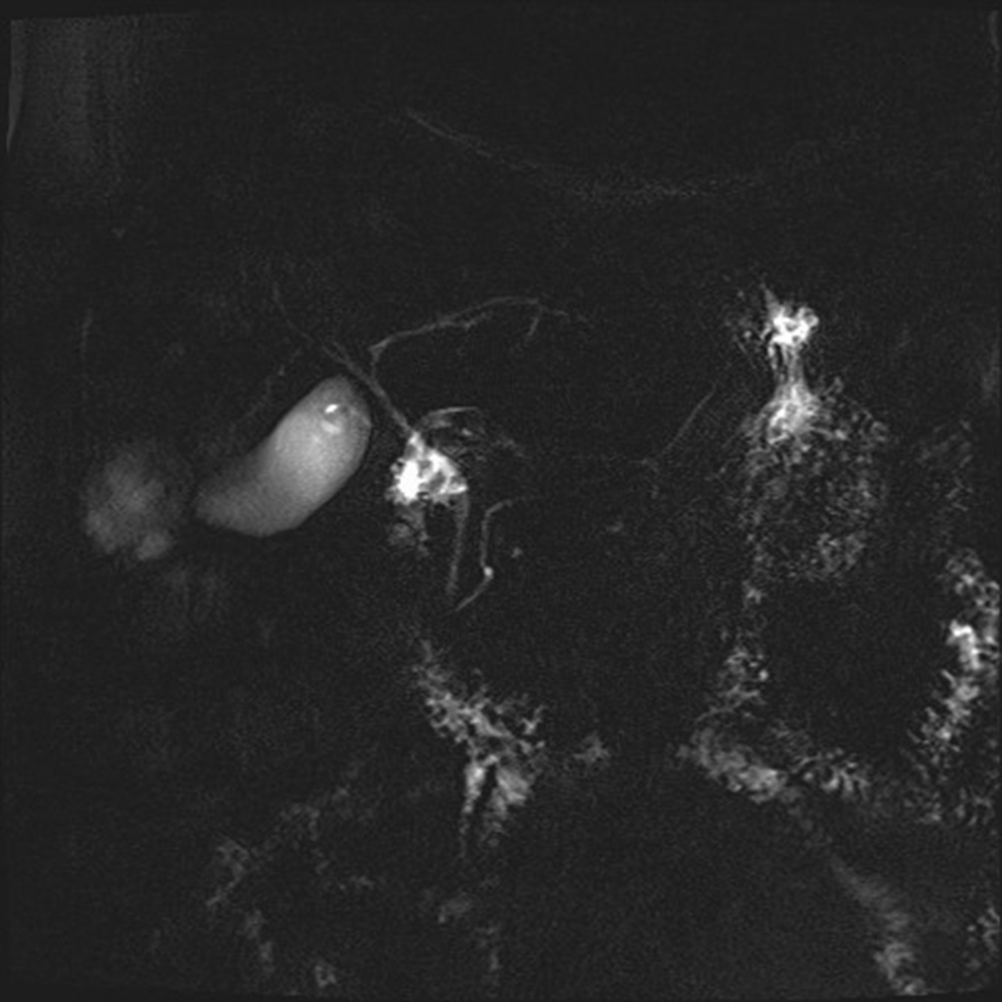
Cholangioresonance sequences.

**Figure 4.**
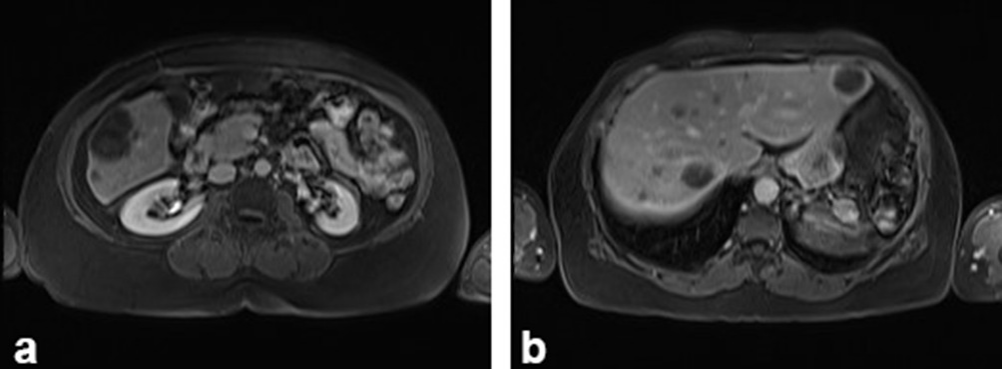
(a) MRI axial *T*_1_ late phase, 5 min after administration of contrast medium, showing low uptake of the contrast medium, which is heterogeneous; central sinus is seen in some lesions and peripheral in others. (b) MRI axial *T*_1_ late phase, 5 min. Lesions show little contrast medium uptake, simulating lesions of a cystic nature.

**Figure 5.**
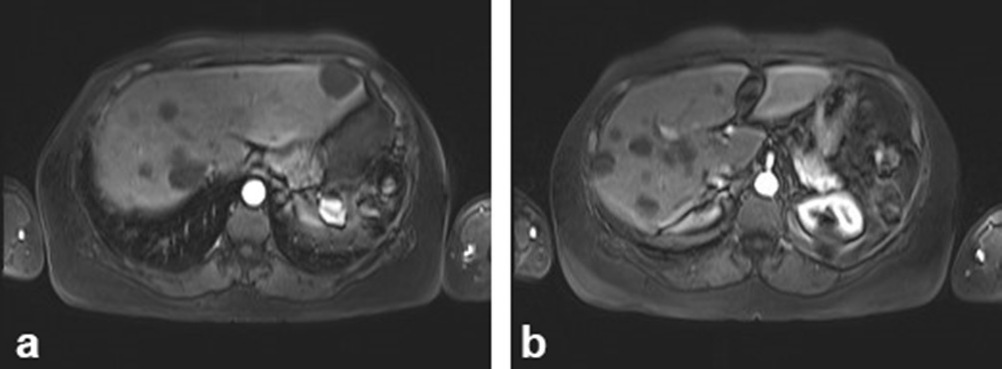
(a, b) MRI axial, arterial phase: multiple low-intensity lesions are identified that do not show contrast medium uptake.

**Figure 6.**
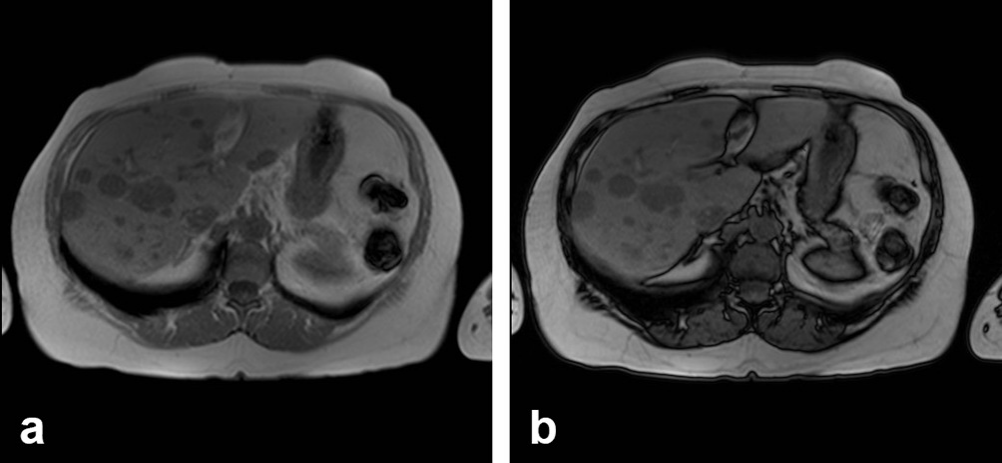
(a and b) Phase and out-of-phase sequences were performed without identifying change in signal intensity, suggesting microscopic fat content in the lesions.

The MRI findings (Figure 2) showed a liver of normal size and morphology, with multiple focal lesions distributed among all segments of the hepatic parenchyma, with variable sizes ranging from a few millimetres to several centimetres, the biggest lesion (in the right lobe) being 5 cm. These lesions appeared hyperintense in *T*_2_ sequences and hypointense in *T*_1_ sequences, without identification of a fatty component in their interior. Predominant restriction in the periphery was observed in diffusion sequences. After contrast administration, some lesions showed discrete heterogeneous peripheral enhancement, whereas others showed intralesional nodular enhancement. Cholangioresonance sequences were performed with results in normal ranges (Figure 3). The lesions present very little contrast uptake even in the late 5-min sequences. This uptake is heterogeneous, being central in some lesions and peripheral in others (Figure 4). Finally, in the MRI in the arterial phase, multiple low-intensity lesions are identified that do not show contrast media uptake (Figure 5). Phase and out-of-phase sequences were performed without identifying change in intensity signal, suggesting microscopic fat content in the lesions (Figure 6).

**Figure 7.**
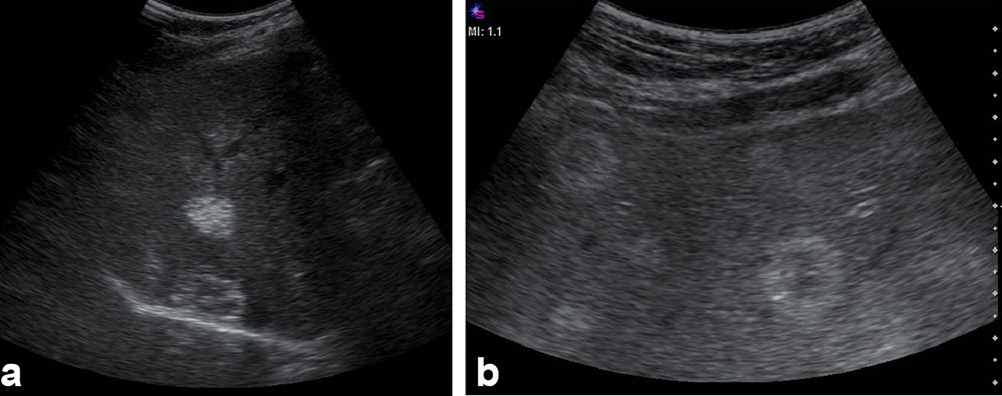
Liver Ultrasound: (a and b) Multiple heterogeneous, predominantly echogenic hepatic lesions were identified, distributed among all liver segments. Some had target-like or annular appearance and others had calcifications. Some had a pattern similar to “hailstorm” described in echinococcosis.

This study was complemented with ultrasound examination (Figure 7), which demonstrated solid, frank echogenic lesions with multiple punctate echogenic foci in their interiors explained by the presence of calcifications; some exhibited annular hyperechogic halo with less echogenic centres. No alterations of the biliary tract were identified in the MRI or ultrasound image.

Permeability of the intra-abdominal vascular structures was observed and morphological changes of chronic liver disease or cirrhosis could not be identified. No other intra-abdominal lesions or adenopathies were found.

Paraclinical studies (liver function test, carcinoembryonic antigen, clotting time, complete blood count and platelets) were requested but all results were in normal ranges.

The diagnostic possibilities based on the imaging findings were infectious involvement by echinococcus versus metastatic lesions of an unknown primary tumour.

**Figure 8.**
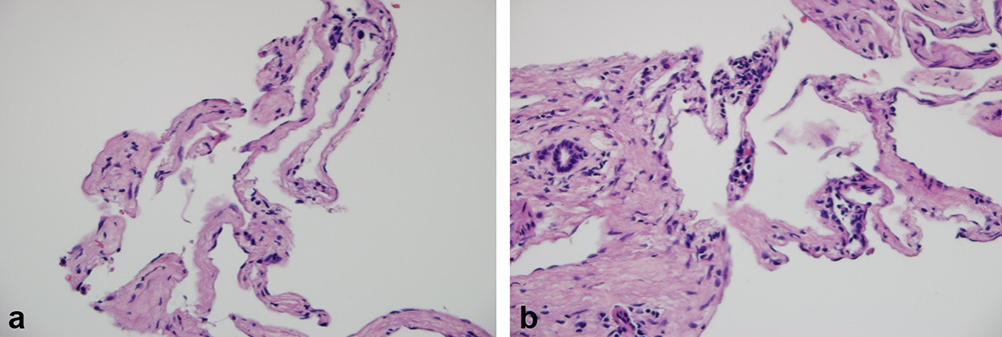
Liver biopsy. (a) Vascular channels lined by endothelial cells without dysplasia, separated by septa of fibrous enlargement (100×); (b) Vascular channels adjacent to a portal space that presents occasional lymphocytes and fibrosis (200×).

An ultrasound-guided biopsy of the dominant lesion located in the right lobule was performed to clarify the diagnosis. A report was obtained within 3 days of the procedure; the pathology findings of the collected liver samples reported “fragments of liver tissue replaced by a benign lesion of vascular origin characterized by the presence of numerous anastomosing vascular channels within it, lined by endothelial cells without atypia, separated by fibrous connective tissue septa with ectatic and obliterated vessels; findings are consistent with sclerosing cavernous haemangioma” (Figure 8).

## Discussion

Hepatic haemangiomas are benign vascular non-neoplastic liver lesions of the hepatic mesenchyme that are well circumscribed and sponge shaped; the majority are congenital and mostly of the cavernous subtype.^1^ Most lesions tend to be smaller than 5 cm and asymptomatic.^1,2^

The prevalence of cavernous hepatic haemangiomas ranges from 1–20%,^2^ being a common lesion in the general population. These haemangiomas are more commonly diagnosed in patients between 30 and 50 years of age and in women relative to men, at a 5:1 ratio.^1^

Most of the lesions are unique; only 10% of cases exhibit multiple lesions.^1^

In case of typical haemangioma, imaging modalities are highly reliable for diagnosis, especially MRI, which has a sensitivity and specificity of greater than 90%. With respect to the appearance of typical haemangiomas in ultrasound, an echogenic, homogeneous mass is evidenced with well-defined margins and posterior acoustic enhancement.^3,4^

In CT, these lesions show low attenuation in the simple phase; after contrast administration, globular discontinuous enhancement occurs peripherally, and this peripheral attenuation is equal to the density of the contrast of the aorta; in the venous and late phase, there is centripetal enhancement that progresses to completely fill the lesion.^1^

In MRI, the lesions present well-defined margins and very high *T*_2_-signal intensity, similar to the cerebrospinal fluid. With administration of contrast medium, specificity increases up to 98% for the characteristic enhancement pattern identical to that described in CT.^1^ In MRI as well as in ultrasound, three imaging patterns have been described:

- Pattern 1—uniform high signal intensity after contrast
- Pattern 2—peripheral nodular enhancement with centripetal progression until uniform signal intensity is reached
- Pattern 3—peripheral nodular enhancement with centripetal progression and a persistent central scar.^5^

Among the atypical haemangiomas reported in the literature, we found the following to have been described: giant, of heterogeneous uptake, rapidly filling, calcified, hyalinized, cystic or multilocular, with liquid-liquid levels and pedunculated. These can be associated with abnormalities underlying the lesion, such as arterioportal shunts, peripheral nodular hyperplasia and capsular shrinkage, which are also present in the diffuse focal lesions of haemangiomatosis.^1–3^

This case presented itself as a diagnostic challenge because multiple lesions, predominantly echogenic, with some being heterogeneous and others having calcifications with target appearance, were evident on ultrasound. The low enhancement and heterogeneity detected in all phases of MRI and CT could be explained by the presence of microcalcifications and/or microinfarctions. The appearance of the haemangiomas in the diffusion images reinforced the annular imaging, and in late post-contrast images the lesions remained almost unchanged with respect to the venous phase.

Regarding differential diagnoses, hydatid disease at ultrasound can present several patterns such as “hailstorm” appearance, pseudocystic, haemangioma-like, ossificans and metastasis-like lesions.^6^ Haemangioma-like hydatid diseases are difficult to distinguish from atypical haemangiomas; they are clearly demarcated but not homogeneous and very often represent a significant diagnostic change.^6^ In this case, the liver lesions had an ultrasound appearance similar to that described in hepatic alveolar echinococcosis as “hailstorm pattern,” which is characterized by hyperechoic lesions with irregular boundaries, non–homogeneous, with or without acoustic shadow.

Hepatic haemangiomas rarely show calcifications. These have been reported in 10% of the ultrasound images and 20% of CT scans.^7^ These can be observed at the marginal or central level of the lesion. Calcified haemangiomas can exhibit poor contrast, especially in the arterial phase of CT or MRI. Therefore, a liver haemangioma should not be ruled out in hepatic lesions that do not enhance in the arterial phase or contain small calcifications.

Liver metastases are another differential diagnosis to consider in this case, especially because there are multiple lesions. These can be classified as hypervascular (enhanced in the post-gadolinium arterial dominant phase) and hypovascular (showing little uptake in the arterial phase and usually presenting enhancement in late contrast images). They can also have calcifications, especially in the case of metastases of mucinous carcinoma of the colon, breast, stomach or ovary as well as in melanoma, thyroid carcinoma, chondrosarcoma, leiomyosarcoma and neuroblastoma. Such calcium deposits can be dotted, amorphous, scaly, granular or poppy-seed-like.^8^

In conclusion, although rare, the possibility of multiple haemangiomas with microcalcification, target appearance and low enhancement can be included in the differential diagnosis of other liver lesions such as metastatic disease and hydatid disease.^9^

With regard to treatment, with asymptomatic atypical cavernous hepatic haemangiomas clinical and imaging follow-up is indicated, preferably in 6 to 12 months, using the same diagnostic modality as that used for diagnosis. But owing to radiation issues, monitoring with MRI is suggested, for the purpose of comparison. If there is no change, further follow-up is not recommended.^2^ In some patients who are symptomatic because of extrinsic compression, haemangiomas have been managed with surgical resection. Non-surgical treatments include hepatic arterial embolization and radiotherapy; however, these are rarely first-choice treatments.^2^

## Learning points

1. Hepatic haemangiomas are benign vascular non-neoplastic liver lesions of the hepatic mesenchyme that are well circumscribed and sponge shaped; the majority are congenital and mostly of the cavernous subtype.^1^ Most lesions tend to be smaller than 5 cm and asymptomatic.
2. Among the atypical haemangiomas reported in the literature, we found the following to have been described: giant, of heterogeneous uptake, rapidly filling, calcified, hyalinized, cystic or multilocular, with liquid-liquid levels and pedunculated.
3. The appearance of the haemangiomas in the diffusion images reinforced the annular imaging and in late post-contrast images the lesions remained almost unchanged with respect to the venous phase.
4. Haemangioma-like hydatid diseases are difficult to distinguish from atypical haemangiomas; they are clearly demarcated but not homogeneous and very often represent a significant diagnostic change.
5. The possibility of multiple haemangiomas with microcalcification, target appearance, and low enhancement can be included in the differential diagnosis of other liver lesions such as metastatic disease and hydatid disease.
6. With regard to treatment, with asymptomatic atypical cavernous hepatic haemangiomas clinical and imaging follow-up is indicated, preferably in 6 to 12 months.
